# Microinjection of angiotensin II into zebrafish embryos induces transient dilation and elastin disruption of the dorsal aorta

**DOI:** 10.14814/phy2.70259

**Published:** 2025-02-24

**Authors:** Shota Tanifuji, Keiko Uchida, Genri Kawahara, Takashi Nakamura, Saki Iida, Yukiko K. Hayashi, Utako Yokoyama

**Affiliations:** ^1^ Department of Physiology Tokyo Medical University Tokyo Japan; ^2^ Department of Pathophysiology Tokyo Medical University Tokyo Japan

**Keywords:** angiotensin II, blood vessels, development, elastic fibers, zebrafish

## Abstract

The effects of angiotensin II (AngII) on blood vessel development and remodeling have been extensively investigated in mice and humans. However, its action on the vessels in the zebrafish remains largely unknown. To investigate whether AngII affects vascular morphology in vivo, we administered AngII into the endothelial‐specific transgenic reporter zebrafish (*Tg[kdrl:EGFP]*) at the 1‐cell stage. The average dorsal aortic diameter of five serial positions was significantly increased by 20% in AngII‐injected zebrafish compared with buffer‐injected controls at 5 days post‐fertilization. Histological studies in AngII‐injected zebrafish at 8 weeks post‐fertilization showed that elastic fiber formation was partly attenuated, with enhanced matrix metalloproteinase‐2 expression in the dorsal aorta without dilation. These results suggest that AngII induced transient aortic expansion in early larvae and may affect vascular elastic fiber formation in adult zebrafish. The use of the AngII‐injected zebrafish model is a potential tool to dissect the mechanisms of disruption of elastic vascular wall formation in the aorta.

## 1 INTRODUCTION

The renin‐angiotensin system is involved in numerous physiological and pathological phenomena, including aortic wall formation. Aortic aneurysm (AA) rupture is fatal and a significant cause of mortality. Angiotensin II (AngII) and the renin‐angiotensin system are implicated in the development of AA. Although significant progress has been made in understanding the molecular basis of aneurysm development and growth, the efficacy and safety of beta‐blockers and AngII receptor blockers on AA remain controversial (Isselbacher et al., 2022).

Studies in genetically engineered mice have demonstrated that AngII, the major effector peptide of the renin‐angiotensin system, plays a critical role in vascular development (Nagata et al., 1996; Niimura et al., 1995), and an excessive level of AngII promotes pathological remodeling such as elastic fiber dysregulation in the large vessels (Forrester et al., 2018; Sawada et al., 2022). AngII has been shown to induce aneurysm formation based on elastic fiber degradation in both wild‐type mice (Rateri et al., 2014) and multiple genetically modified mice, including *ApoE* ^−/−^ mice, *Ldlr* ^−/−^ mice, and vascular smooth muscle cell–selective prostaglandin E receptor 4‐overexpressed mice (Daugherty et al., 2000; Daugherty & Cassis, 1999; Hiromi et al., 2020). AngII infusion increases the activation of nuclear factor‐κB (Tham et al., 2002) and the expression of urokinase‐type plasminogen activator (Wang et al., 2001), which leads to the mobilization of inflammatory cells and subsequent elastic fiber degradation in the vessel wall. AngII increases NADPH‐dependent reactive oxygen species production in vascular smooth muscle cells, thereby inducing the secretion of cyclophilin A and matrix metalloproteinase‐2 (MMP‐2) and elastic fiber degradation in the aorta (Satoh et al., 2009). Emerging evidence indicates that AngII induces inflammatory cell recruitment, reactive oxygen species production, and protease activation in the vessels.

Zebrafish have been widely used in research because of their high genetic homology to humans and the large number of offspring in each generation. It is also an attractive model for studying cardiovascular disease because its physical transparency permits direct microscopic assessment, and medications can be administered into bath water, from which they are systemically absorbed (Eberlein et al., 2021). AngII results in elevation of intracellular calcium in the toadfish (*Halobatrachus didactylus*) dorsal aorta via its receptor, suggesting that the AngII receptor in fish is similar to the AngII type 1 receptor in humans (Qin et al., 1999). AngII in fish, as in humans and rodents, increases arterial pressure and vasoconstriction (Russell et al., 2001). Intradermal administration of AngII to adult zebrafish causes cardiac hypertrophy and cardiac fibrosis, as is also seen in rodents (Daugherty et al., 2000; Daugherty & Cassis, 1999; Hiromi et al., 2020; Joshi et al., 2021; Rateri et al., 2014). However, the effects of AngII on vascular morphogenesis and remodeling in zebrafish remain incompletely elucidated (Joshi et al., 2021; Russell et al., 2001), though monitoring the morphology of the dorsal aorta in early larvae may be useful for high‐throughput screening to discover drugs that inhibit AngII‐mediated vascular dysregulation. In this study, we aimed to investigate the effect of AngII on vascular morphology and elastic fiber formation during development in zebrafish.

## 2 MATERIALS AND METHODS

### 2.1 Animals and angiotensin II injection

A transgenic reporter zebrafish line *Tg(kdrl:EGFP)* (Choi et al., 2007), which has a vascular endothelium‐specific *kdrl* promoter directing EGFP (enhanced green fluorescent protein) expression, was obtained from the Aquatic Resources Program, Boston Children's Hospital (Boston, MA, USA). They were cultured at 28.5°C according to standard procedures (Nusslein‐Volhard & Dahm, 2002) and standard criteria (Kimmel et al., 1995). This study was carried out in compliance with the ARRIVE guidelines and in accordance with the National Institutes of Health *Guidelines for the Care and Use of Laboratory Animals*.

AngII (Sigma‐Aldrich, St. Louis, MO, USA) was prepared at a concentration of 40 mg/mL in distilled water. Forty nanograms of AngII in 1 nL of distilled water were prepared and injected 2 nL or 4 nL into the yolk of 1‐cell stage *Tg(kdrl:EGFP)* embryos using an electric microinjector IM‐31 (Narishige, Tokyo, Japan). Distilled water (4 nL) was used as a control.

### 2.2 Imaging and quantification of the dorsal aorta diameter

**FIGURE 1:**
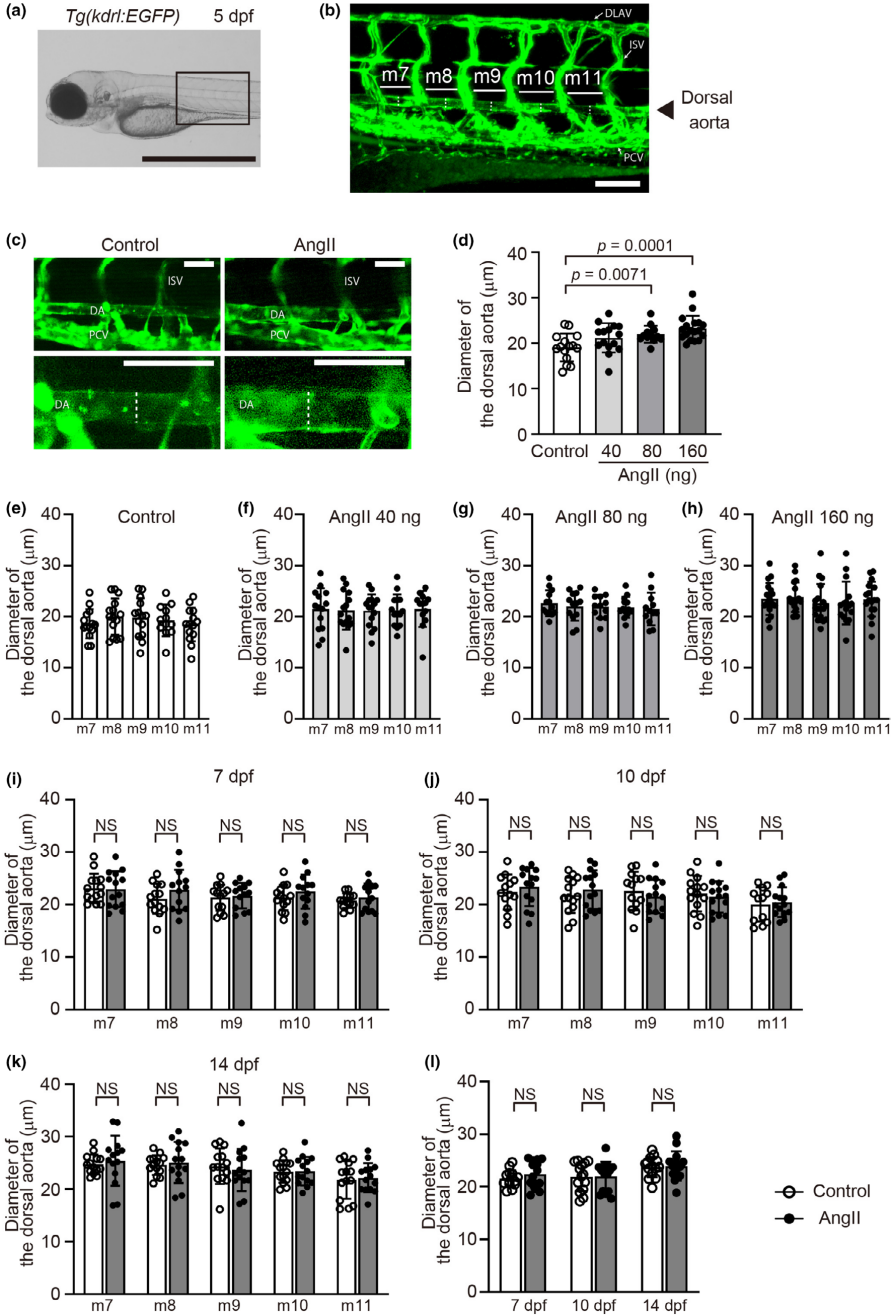
Angiotensin II (AngII) administration increased the dorsal aorta diameter in zebrafish embryos. (a) A lateral bright field image of *Tg(kdrl:EGFP)* control embryos at 5 days post‐fertilization (dpf). Scale bar: 1 mm. (b) A confocal microscopy image of the EGFP‐positive blood vessels of the boxed region in (a); m7 to m11 indicates myotomes 7–11, respectively. Scale bar: 100 μm. (c) Representative lateral dorsal aorta images of control or AngII (160 ng)‐injected *Tg(kdrl:EGFP)* embryos at 5 dpf. The lower panels are higher‐magnification images of the upper panels. Scale bars: 50 μm. (d) Quantification of the average diameter of the dorsal aorta. (e–h) Dorsal aorta diameter at myotomes 7–11 in control or AngII (40 ng, 80 ng, or 160 ng)‐injected *Tg(kdrl:EGFP)* embryos at 5 dpf. *n* = 15 (control), 15 (40 ng), 13 (80 ng), and 18 (160 ng). (i–k) Dorsal aorta diameter at myotomes 7–11 in control or AngII (160 ng)‐injected *Tg(kdrl:EGFP)* embryos at 7 dpf (i), 10 dpf (j), and 14 dpf (k). (l) Quantification of the average diameter of the five serial segments of the dorsal aorta. *n* = 14 (control, white), and *n* = 14 (AngII 160 ng, gray). NS indicates not significant. Dotted lines in (b) and (c) indicate the diameter of the dorsal aortae. DA, dorsal aorta; DLAV, dorsal longitudinal anastomotic vessel; ISV, intersegmental vessel; PCV, posterior cardinal vein.

After injection of AngII or distilled water into 1‐cell stage *Tg(kdrl:EGFP)* embryos, 5, 7, 10, and 14 days post‐fertilization (dpf) embryos were anesthetized with tricaine (E10521, Sigma‐Aldrich, St. Louis, MO, USA) solution and positioned in 3% methylcellulose (M0387, Sigma‐Aldrich, St. Louis, MO, USA). The dorsal aortas in live embryos were imaged at a resolution of 0.41 μm/pixel using an LSM710 confocal microscope (Plan‐Apochromat 20x/0.8M27, ZEN system 2010 software ver. 6,0,0,485, Zeiss, Oberkochen, Germany). Aortic diameters at five serial segments in the region behind the swim bladder were independently measured by two scientists using ImageJ software (version 1.53a, National Institutes of Health, Bethesda, MD, USA; Figure 1a,b). The average diameter of the five serial segments was used for quantification.

### 2.3 Tissue staining and immunohistochemistry

*Tg(kdrl:EGFP)* zebrafish were used to investigate the development of elastic lamellae in the dorsal aorta at 4 weeks post‐fertilization (wpf), 6 wpf, and 8 wpf. All zebrafish in the experiment at 4 wpf were sexually indistinguishable. The experiment with adult zebrafish included seven zebrafish: two females and one male at 6 wpf, and two females and two males at 8 wpf. To investigate the chronic effect of AngII administration at the 1‐cell stage embryos, eight AngII‐injected *Tg(kdrl:EGFP)* zebrafish (four females and four males) and five distilled water–injected *Tg(kdrl:EGFP)* zebrafish (three females and two males) were used at 8 wpf.

Zebrafish were fixed in 10% neutral buffered formalin overnight at 4°C and stored in 70% ethanol for 24 h at 4°C. Paraffin‐embedded blocks containing zebrafish were cut into 4‐μm‐thick transverse sections and placed on glass slides. Tissue sections were stained with Elastica van Gieson according to the manufacturer's instructions (40321, Muto Pure Chemicals, Tokyo, Japan) to quantify maximum aorta diameter and vascular elastic fiber formation. The dorsal aorta between the rostral end of the swim bladder and the caudal end of the intestine was independently evaluated by two scientists using ImageJ software. The aortic diameter was calculated using the values of the aortic perimeter. The area of the tunica media was determined by subtracting the lumen area from the cross‐sectional area of the dorsal aorta. Elastic fiber degradation was graded as described previously (Hiromi et al., 2020; Sun et al., 2007). Briefly, grade 1 indicates normal elastic fibers, and grades 2 through 4 indicate elastic lamellae with mild‐to‐severe degradation. Tissue sections were also stained with Masson's trichrome (HT15‐1KT, Sigma‐Aldrich, St. Louis, MO, USA), Picrosirius red (24901‐250, Polysciences, Warrington, PA, USA), and Alcian blue (20124, Muto Pure Chemicals, Tokyo, Japan) according to the manufacturer's instructions to estimate extracellular matrix, including collagen and proteoglycan. The sections stained with Picrosirius red were imaged under polarized light to evaluate type I and III collagen deposition.

Immunohistochemistry was performed using primary antibodies for procollagen type I (SP1.D8; Developmental Studies Hybridoma Bank, Iowa City, IA, USA), aggrecan (MA3‐16888 (BC‐3); Thermo Fisher Scientific, Waltham, MA, USA), L‐plastin (ab210099; Abcam, Cambridge, UK) and MMP‐2 (AF1488; R&D Systems, Minneapolis, MN, USA). Biotinylated mouse, rabbit, or goat antibody (Vectastain Elite ABC IgG kit; PK‐6102, Vector Labs, Burlingame, CA, USA) was used as the secondary antibody, and the presence of targeted proteins was determined using 3,3′‐diaminobenzidine (k3466, DAKO, Glostrup, Denmark). Staining specificity was confirmed by the omission of the primary antibodies. The slides were counterstained with Mayer's hematoxylin.

### 2.4 Statistical analysis

**FIGURE 2:**
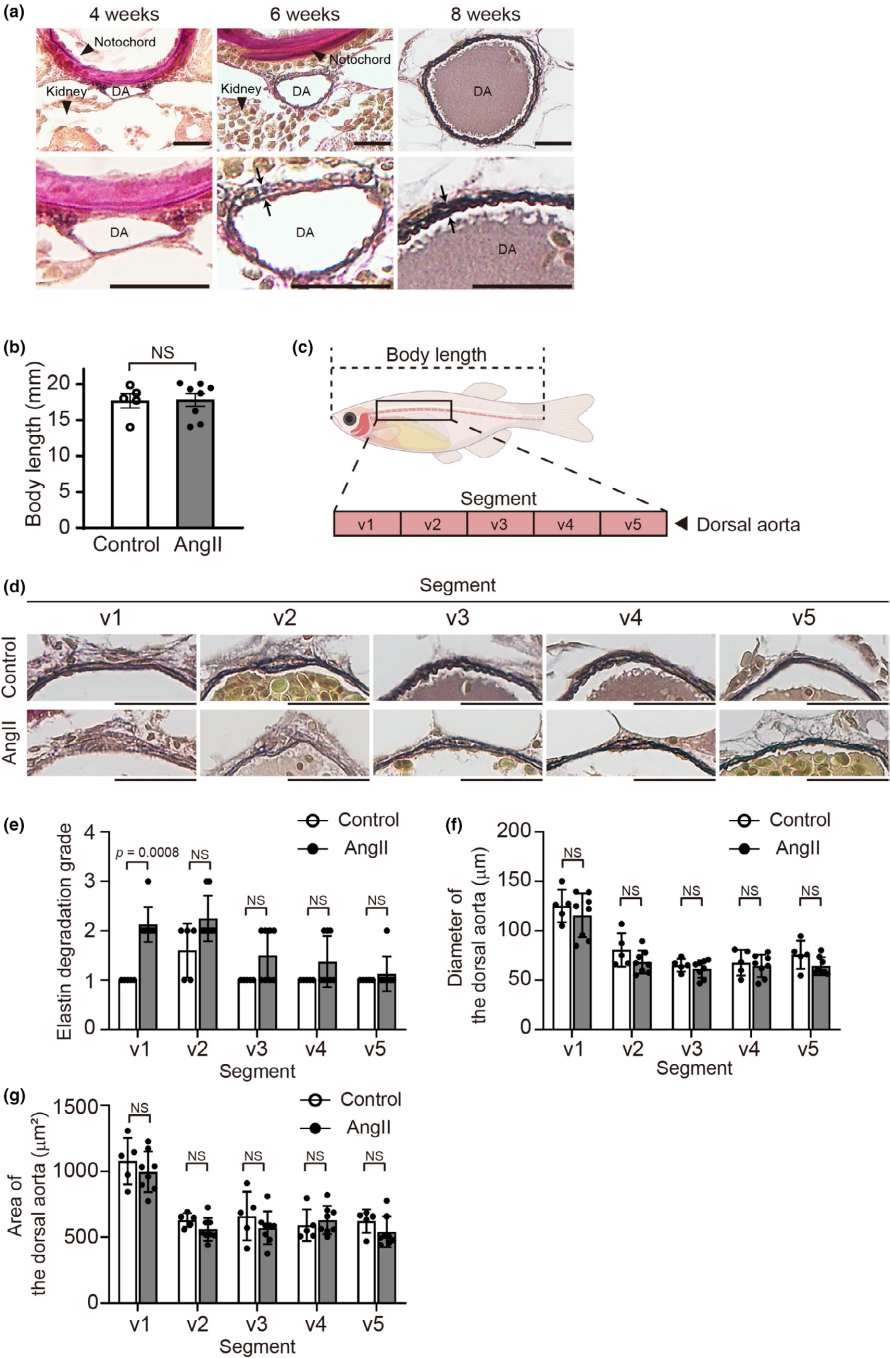
Angiotensin II (AngII) attenuated elastic fiber formation in the adult zebrafish dorsal aorta. (a) Representative images of Elastica van Gieson–stained transverse sections of the dorsal aorta of *Tg(kdrl:EGFP)* zebrafish at 4, 6, and 8 weeks post‐fertilization (wpf) (The experiment with adult zebrafish included seven zebrafish: Two females and one male at 6 wpf, and two females and two males at 8 wpf. *n* = 2, 3 (two females and one male), and 4 (two females and two males), respectively). Lower panels indicate higher magnification views of the dorsal aorta in the upper panels. 2 to 3 elastic lamellae were seen at 8 wpf (showed by arrows). DA, dorsal aorta. Scale bars: 20 μm. (b) Body length, length from the tip of the snout to the base of the tail, of control and 160 ng AngII‐injected *Tg(kdrl:EGFP)* zebrafish at 8 wpf. *n* = 5 (control) and 8 (160 ng AngII‐injected zebrafish). NS indicates not significant. (c) The illustration indicates the dorsal aorta region, that is, segments v1 through v5, for quantitative analyses. (d) Representative Elastica van Gieson–stained transverse images of the dorsal aorta segments v1 to v5 at 8 wpf. Scale bars: 20 μm. (e–g) Quantification of elastin grading, aorta diameter, and area of the segments v1 to v5 in (d). *n* = 5 (control) and 8 (160 ng AngII‐injected zebrafish). NS indicates not significant.

All values are shown as the mean ± standard deviation of more than three independent experiments. A Kruskal–Wallis test followed by Fisher's least significant difference post hoc test and Mann–Whitney *U* test was used to analyze the data of Figure 1d. A Mann–Whitney *U* test was used for Figures 1i–l and 2b,e–g. A value of *p* < 0.05 was considered statistically significant.

## 3 RESULTS

### 3.1 AngII administration increased the diameter of the dorsal aorta in *Tg(kdrl:EGFP)* zebrafish embryos

To investigate AngII‐induced morphological changes in the early larvae dorsal aorta, we microinjected AngII into 1‐cell stage embryos of *Tg(kdrl:EGFP)* zebrafish, in which endothelial cells are labeled with EGFP. The dorsal aorta morphology of zebrafish at 5 dpf was monitored by EGFP detection (Figure 1a,b). We measured the diameter of the dorsal aorta ranging in the region from myotome 7 to 11, in which EGFP‐positive vasculature can be detected (Figure 1b), and found that AngII at a dose of 80 ng or 160 ng per embryo significantly increased the average dorsal aorta diameter (Figure 1c,d), though it was reported that dorsal aorta diameter physiologically decreases towards the first 6 days in larval zebrafish (Bagatto & Burggren, 2006). AngII‐induced aortic dilation was evenly observed through five serial myotomes (Figure 1e–h) and was not associated with the distance from the intersegmental vessels, as seen in Figure 1c. The dorsal aorta diameters were no longer significantly different between the AngII‐injected group and controls from 7 dpf onwards, regardless of their location (Figure 1i–l).

### 3.2 Elastic fiber dysregulation in the dorsal aorta of AngII‐injected adult zebrafish

The involvement of excessive AngII in elastic fiber dysregulation has been recognized previously in mice and humans (Forrester et al., 2018; Sawada et al., 2022). We investigated elastic fiber development in the zebrafish dorsal aorta. Following Elastica van Gieson staining, no visible elastic fiber was observed at 4 wpf (Figure 2a). A single elastic lamella was visible at 6 wpf, and 2 to 3 elastic lamellae were detected at 8 wpf (Figure 2a), suggesting that vascular elastic lamella fabrication was completed and could be analyzed in the zebrafish dorsal aorta at 8 wpf. Based on these results, we used zebrafish at 8 wpf to assess elastic fiber formation.

We investigated the effect of AngII administration in 1‐cell stage embryos on elastic fiber formation in the dorsal aorta of *Tg(kdrl:EGFP)* zebrafish. AngII injection at the 1‐cell stage did not affect body length at 8 wpf (Figure 2b). We assessed the dorsal aorta in the region from the rostral end of the swim bladder to the caudal end of the intestine, in which the region was divided into five parts, v1 through v5 (Figure 2c). Elastin degradation grade was significantly greater in AngII‐injected zebrafish dorsal aorta compared with the control in segment v1. A similar trend was observed in segment v2, although it did not reach statistical significance (Figure 2d,e). The diameter and area of the dorsal aorta at each segment did not differ between the control and the AngII‐injected groups (Figure 2f,g).

### 3.3 AngII administration increased MMP‐2 expression in the dorsal aorta of adult zebrafish

**FIGURE 3:**
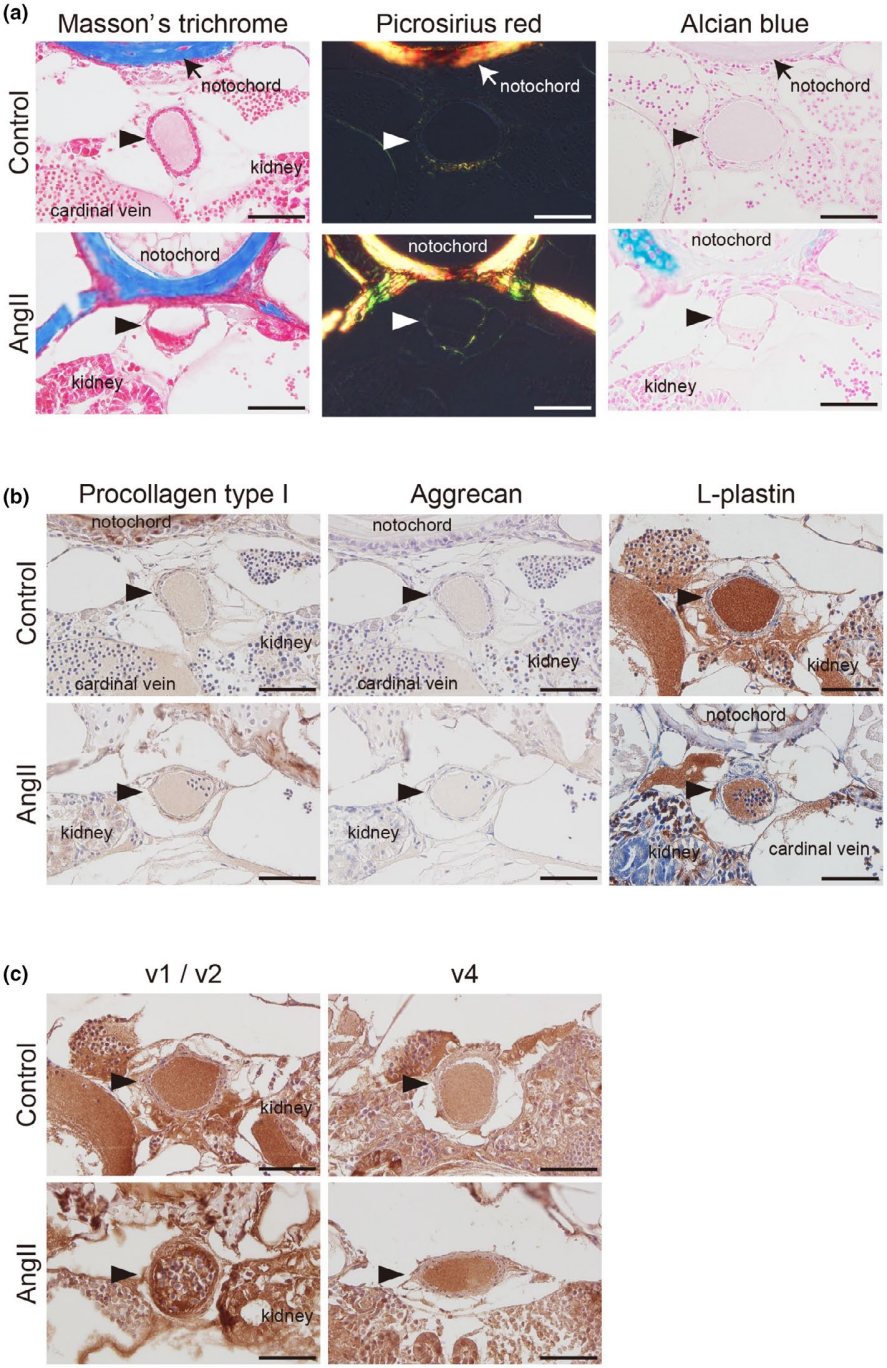
Matrix metalloproteinase (MMP)‐2 upregulation in the dorsal aorta of angiotensin II (AngII)‐injected zebrafish. Representative Masson's trichrome, Picrosirius red, and Alcian blue‐stained (a), immunohistochemically stained with anti‐procollagen type I, aggrecan, and L‐plastin antibodies (b) of the transverse sections taken from the segments v1/v2 of the dorsal aorta in control and 160 ng AngII‐injected *Tg(kdrl:EGFP)* zebrafish at 8 wpf. (c) Representative immunohistochemically stained with anti‐MMP‐2 antibody of the transverse sections taken from the rostral (v1 or v2) and caudal (v4) segments of the dorsal aorta in control and 160 ng AngII‐injected *Tg(kdrl:EGFP)* zebrafish at 8 wpf. Arrowheads indicate the dorsal aorta. Scale bars: 50 μm.

Elastic fiber dysregulation is often accompanied by remodeling of the extracellular matrix, such as collagen and proteoglycans. We conducted Masson's trichrome staining, Picrosirius red staining, Alcian blue staining, and immunohistochemistry for collagen type I and aggrecan. We observed relatively weak signals and no region‐specific aberrant accumulation of collagen and proteoglycans in both the AngII‐injected group and controls (Figure 3a,b). Although the area of the dorsal aorta did not differ between AngII‐injected and control zebrafish, the parts of the vascular walls with attenuated elastic fiber formation were thickened, and cells appeared proliferative at segments v1 and v2 in AngII‐injected zebrafish (Figure 2d). Though it was previously reported that AngII administration promotes immune cell infiltration into the vascular wall in mice (Alvarez et al., 2004; Hiromi et al., 2020), we found no L‐plastin‐positive leukocytes in the dorsal aorta of the AngII‐injected zebrafish (Figure 3b, right panels). MMP‐2 promotes elastic fiber degradation in the vessels (Longo et al., 2002). A strong immunoreaction against MMP‐2 was detected in the rostral segments v1 or v2 of the dorsal aortae containing the area of cell proliferation in AngII‐injected zebrafish compared with the controls (Figure 3c, left panels). In contrast, in the more caudal regions (v4), where no elastic fiber changes were observed in either the AngII‐injected or control group (Figure 2d,e), MMP‐2 staining intensity in the two groups was lower than in the AngII‐injected v1 region (Figure 3c right panels).

## 4 DISCUSSION

We demonstrated that AngII administration to 1‐cell stage embryos induced transient aortic expansion at 5 dpf, which returned to normal at 7 dpf, and that attenuated elastic fiber formation in the dorsal aorta with enhanced MMP‐2 production was observed in AngII‐injected zebrafish at 8 wpf. Although AngII‐induced aortic expansion was transient and not associated with aortic aneurysm or dilation in juvenile/adult zebrafish, these data suggest the possibility that excessive AngII during the early embryonic stage is related to the attenuation of vascular elastic lamella development.

In the present study, injection of AngII induced transient aortic dilation in larvae and elastic fiber dysregulation in adult zebrafish. Our findings were comparable to those of Folkesson et al., who showed dorsal aortic dilatation by AngII in early larvae (Folkesson et al., 2016). Alpha smooth muscle actin–positive mural cells begin to cover the zebrafish dorsal aorta at around 4 dpf (Santoro et al., 2009; Whitesell et al., 2014), and the structure and the components of the basement membrane of the dorsal aorta were detected even in 5 dpf. Mural cell coverage of the dorsal aorta and basement membrane assembly are thought to restrict aortic diameter (Stratman et al., 2017). Because the appropriate antibodies for zebrafish smooth muscle cell markers, including α‐smooth muscle actin and transgelin, are not commercially available, vascular mural cell‐targeted reporter zebrafish should be used to evaluate the specific molecules involved in AngII‐induced aortic dysregulation in future studies.

In the AngII injection group, increased MMP‐2 expression was observed in the sections derived from the segment v1 or v2 of the adult dorsal aorta where elastic fibers were fragmented. In contrast, MMP‐2 expression levels in the more caudal regions of the dorsal aorta, which exhibited no elastic fiber abnormalities, were comparable to those in controls. Previous studies demonstrated that AngII promotes MMP‐2 production in endothelial cells (Arenas et al., 2004) and vascular smooth muscle cells (Luchtefeld et al., 2005; Pons et al., 2011). Although AngII may contribute to elastic fiber fragmentation via MMP‐2 upregulation, we were unable to assess MMP‐2 activity due to limitations in our zebrafish experimental system. Future studies are needed to clarify the causal relationship between elastic fiber fragmentation and MMP‐2 activation.

Histological study revealed that a single elastic lamella dividing endothelial and smooth muscle cells can be detected in the dorsal aorta at 6 wpf, and 2 to 3 layers of elastic lamella were fabricated in zebrafish at 8 wpf, suggesting that elastic lamellae fabrication was completed between 1 and 2 months of age in zebrafish. These data are consistent with a previous report showing that no elastic lamella was observed in the dorsal aorta in 7 dpf and 1‐month‐old zebrafish, and 2 to 3 elastic lamellae existed in 3‐month‐old adult zebrafish (Miano et al., 2006). In mouse aorta, the elastic lamellae begin to form around embryonic day 15 (Davis, 1995); the elastic lamellae in the zebrafish dorsal aorta were detected much later, although both zebrafish and mice reach sexual maturity and are considered adults by ∼3 months. Mechanical forces generated by blood flow and blood pressure profoundly affect vascular elastic fiber formation (Wolinsky & Glagov, 1967). Because systolic blood pressure is much lower (∼5 mmHg) in adult zebrafish (Hu et al., 2001) than in adult mice (approximately 130 mmHg) (Tiemann et al., 2003), the elastic lamella fabrication in zebrafish that occurs later in the development period may be attributed to the smaller diameter of the dorsal aorta and lower pressure of the circulatory system.

A decrease in aortic elasticity has been reported to affect cardiovascular function (Cavalcante et al., 2011). AngII‐injected adult zebrafish developed normally, as shown in Figure 2b, and did not exhibit cardiac failure, edema, or loss of physical activity at least up to 8 weeks of age. AngII‐induced partial elastic fiber fragmentation in the dorsal aorta did not result in overt cardiovascular dysfunction, at least in our study.

Our data demonstrated that elastic fiber dysregulation was detected at segments v1 and v2, the more rostral sides of the adult dorsal aorta. It was possible that AngII might induce elastic fiber dysregulation in the region affected by cardiac output–generated mechanical forces, though the effect of AngII administration on cardiac function in larval zebrafish was not evaluated. Moreover, the region of elastic fiber dysregulation in the dorsal aorta of AngII‐injected zebrafish exhibited increased MMP‐2 expression. Recent advances in cardiovascular imaging have highlighted shear stress as a key etiological factor in aortic dilation (Soulat et al., 2022). Although we were unable to determine the exact reason why elastic fiber dysregulation was observed predominantly in the more rostral regions of the adult dorsal aorta, we speculate that mechanical forces generated by cardiac output may exert a greater influence on AngII‐mediated elastic fiber dysregulation in the rostral region than in the peripheral regions. Additionally, we acknowledge the technical limitations of our experiment. The detection of elastic fiber dysregulation may have been more pronounced in segments v1 and v2 due to structural differences in elastic fiber assembly in the dorsal aorta. The rostral region contains at least two well‐developed elastic laminae, whereas the peripheral regions have thinner and less distinct elastic fibers. As a result, we may have underestimated the effects of AngII on the caudal segments. It was difficult to determine whether the location of the dilated dorsal aorta at 5 dpf corresponded to the region with poorly formed elastic fibers at 8 wpf. Indeed, it is unclear whether AngII‐induced transient aortic dilation is directly linked to inadequate formation of elastic fibers. Because it is unlikely that a single injection of AngII in 1‐cell stage embryos directly affected the aortic wall in adult zebrafish, it is speculated that the dorsal aorta administered with AngII in the early larval stage may become more susceptible to mechanical force during development.

Recently, several papers have reported that transient AngII exposure in mice modulates epigenetic regulation in vascular smooth muscle cells (Greenway et al., 2020; Pothen et al., 2022; Zhang et al., 2023). Further study is warranted to clarify the effect of AngII on endothelial cells, the basement membrane, and mural and smooth muscle cells in early larvae and adults using cell type‐specific genetically modified zebrafish and electron microscopic analyses.

Our observations suggest that AngII administration to 1‐cell stage zebrafish embryos induces aortic expansion in early larvae, which may relate to elastic fiber dysregulation in the wall of the dorsal aorta without aortic dilation in adult zebrafish. AngII‐injected zebrafish would be used as a tool for dissecting the pathogenesis of aortic disorders.

## AUTHOR CONTRIBUTIONS

ST and UY contributed to the conception and experimental design. ST, GK, TN, and SI performed the experiments. ST, KU, GK, TN, SI, YH, and UY analyzed the data. ST, KU, and UY wrote the original draft of the manuscript, and all authors reviewed the manuscript. All authors read and approved the final manuscript.

## FUNDING INFORMATION

This work was supported by MEXT/JSPS KAKENHI (UY, JP24K02427, JP23K18320; ST, JP23K05605, JP20K17730; GK, JP23K06876), the Japan Agency for Medical Research and Development (AMED) (UY, 23ek0210183), and the Intramural Research Grant (2‐5) for Neurological and Psychiatric Disorders of NCNP (YH). This work was supported in part by research support from the Center for Diversity at Tokyo Medical University (TMUCD‐202205).

## CONFLICT OF INTEREST STATEMENT

The authors declare that there are no conflicts of interest.

## ETHICS STATEMENT

The experiments in this study were approved by the Institutional Animal Care and Use Committee of Tokyo Medical University (approval number: R4‐072, R5‐074, and R6‐055).

## Data Availability

The datasets used and analyzed during the current study are available from the corresponding author upon reasonable request.
